# Deaf and Hard-of-Hearing Learners in Emergency Medicine

**DOI:** 10.5811/westjem.2018.8.38550

**Published:** 2018-10-10

**Authors:** Lisa M. Meeks, Alina Engelman, Alicia Booth, Michael Argenyi

**Affiliations:** * University of Michigan, Department of Family Medicine, Ann Arbor, Michigan; † California State University, East Bay, Department of Health Sciences, Hayward, California; ‡ Designated Interpreters LLC, New York, New York; § University of Massachusetts Medical School, Department of Family Medicine and Community Health, Worcester, Massachusetts

## Abstract

Approximately 23% of Americans over age 12 have some level of hearing loss.^1^ Emergency departments can reduce healthcare barriers for deaf and hard-of-hearing (DHoH) patients through improved patient-physician communication. DHoH students, once they become physicians, may provide one mechanism for reducing existing healthcare disparities and communication barriers for DHoH patients, and may be more adept with patients facing other communication barriers. A renewed interest in disability access and a commitment to social justice has increased efforts toward the inclusion of individuals with disabilities in medical education and training. Despite this increased interest and a growing number of DHoH students entering medical education, DHoH students continue to be dissuaded from specialty careers such as emergency medicine (EM) over concerns regarding effective communication and ability. Given the academic medicine communities’ commitment to diversity, a recounting of the successful inclusion of DHoH students in EM can benefit medical education and practice.

In this account, the authors reflect on the successful experiences of a visiting DHoH medical student in an academic EM rotation at a Level I trauma hospital that serves a diverse population, and they identify the potential challenges for DHoH students in an EM setting, offer solutions including reasonable accommodations, and provide commentary on the legal requirements for providing full and equal access for DHoH students. We secured permission from the student to share the contents of this article prior to publication.

## INTRODUCTION

Deaf and hard-of-hearing (DHoH) individuals* over the age of 12 comprise 23% of the U.S. population,^1^ and over 500,000 patients use American Sign Language (ASL).^2^ Disproportionate to the general population, allopathic medical students with disabilities account for only 2.7% (1,547) of the total medical student population and only a fraction of these (38) are DHoH students.^3^ Medical schools may unintentionally discourage DHoH students from entering specialties such as surgery, obstetrics and gynecology (OB/GYN) or emergency medicine (EM) given the lack of knowledge regarding this population and the false belief that accommodations are not possible, too complicated, too costly, or that trainees are simply unable to perform the duties of a physician. A recent paper suggests that students with disabilities self-report being counseled out of subspecialties such as surgery, OB/GYN, and EM,^4^ while a 2013 study shows that the majority of DHoH physicians (68%) practice in primary care specialties, supporting the idea that the majority of DHoH physicians do not enter subspecialities.^5^ It may be that experiences in medical school and visiting rotations negatively inform students’ choices to forgo these specialties. Despite a growing interest in the experiences of DHoH students, there remains a dearth of information about the experiences of this population in subspecialty electives such as surgery, OB/GYN and EM. To our knowledge only one article exists that discusses a DHoH student’s experiences in an anesthesia rotation.^6^

Researchers suggest that the inclusion of DHoH students, residents and physicians in the medical education continuum could offer multiple benefits to peers and patients alike including increasing disability awareness, improving interactions with DHoH patients and family members;^7^,^8^ building empathy for persons with disabilities;^9^ and promoting an accessible and supportive environment for patients and physicians, including aging physicians who experience hearing loss as part of natural aging.^8^ DHoH patients may benefit from improvements in knowledge, attitudes, and communication that results from teaching medical students how to work with interpreters^9^ specifically in emergency department (ED) settings where communication is central to patient outcomes. This is especially relevant for the DHoH population that uses ASL, as these patients are more likely to use the ED, when compared to the general hearing population.^10^ Disparities in healthcare and poorer outcomes exist for DHoH patients.^11^–^13^ Language-concordant patient-providers fluent in ASL may help reduce these disparities. For example, a 2011 study showed that ASL users who received primary care from ASL-using physicians were more likely to use preventive services.^14^ It may be that physicians skilled at creatively navigating diverse and alternative forms of communication are able to provide more informed care to DHoH patients.^7^,^15^

While reduced healthcare disparities for patients and a commitment to social justice should drive the inclusion of DHoH students in medicine, recent court decisions have supported qualified DHoH individuals in the healthcare workforce noting that DHoH individuals are appropriate providers when properly accommodated.^16^–^19^ Despite the courts’ support of DHoH students and employees, and the greater focus on diversity and inclusion in medical education, there remains a great deal of stigma for DHoH individuals in medicine.^20^,^21^ For example, concern has been expressed regarding effective communication with DHoH students. However, communication between non-DHoH physicians and teams is of equal concern in medicine. Techniques including establishing set protocols, using a check-back process to verify communication, and communicating the plan to the team members have proven effective in reducing communication errors in EM.^22^ The same recommendations that guide hearing physicians also allow DHoH students to operate within a team and to provide excellent care to their patients. The addition of DHoH students in the ED may reduce common errors among *all* physicians through (1) a focus on accurate translation,^23^ (2) patient care diversity awareness,^24^ and (3) improved access to care through increased cultural competency in working with the DHoH population.^25^

### Case Report on Deaf Student in Emergency Medicine

A deaf medical student completed a one-month visiting rotation in EM at a medical school in the Western U.S. The student had a history of using hearing aids, cochlear implants, communication access real-time transcription (CART), Cued Speech transliteration, and ASL interpreters (Table). With appropriate accommodations, the student performed well in undergraduate and graduate school. The student used designated healthcare interpreters (DI) – sign language interpreters linguistically specialized in working with healthcare professionals – throughout the clinical years in medical school and during the visiting EM rotation. The DIs were provided by the student’s home institution who maintained financial responsibility for the interpreting services and full access for the student’s educational experience.

### Application and Disclosure of Disability

The student applied to the EM rotation through the Visiting Student Application Service. Once accepted, and two months prior to the start of the rotation, the student notified the school of the need for accommodations. The student’s designated interpreter contacted the institution’s Americans with Disabilities Act (ADA) designee to request accommodations and to provide guidelines and guidance for working with a deaf student. Two weeks prior to the start of the program, the program director and disability director provided a brief educational outreach to the ED staff, including techniques for working with deaf students in the clinical setting. The student and DI were invited to share their insights about working in the department at the conclusion of the rotation.

The ED setting presents challenges for all students, specifically a fast-paced and stressful working environment, interacting with patients speaking multiple languages, tight and noisy working spaces, witnessing trauma and overall loss of control in emergency situations. Yet the deaf student’s feedback about the rotation was positive. The student and the DI noted the inclusiveness of the experience in this environment, including a respectful, responsive and communicative team. For example, hospital staff directly approached the student, not the DI, when they had questions about communication (e.g., inquiries about the amplified stethoscope). Educational materials and experiences for students in the program were equally accessible for the deaf student, and the program expressed genuine interest and excitement regarding the diversity the deaf student brought to their program.

### Mechanisms for Inclusion

The program director welcomed the student and set clear expectations for the ED team. The DI was included in every interaction from orientation to patient care. Access to orientation items and to the virtual learning platform were completely accessible as a result of being addressed proactively with the program director, student coordinator, disability services office, and designated interpreter. By requesting accommodations and accessible materials two months in advance, the student ensured 1) the addition of captioning to instructional videos contained in online learning platforms, 2) complete scheduling of the DI for didactic and clinical activities, and 3) the development of specialized medical sign language for the rotation (for terminology not currently designated in ASL) in advance of the student’s arrival. This collaborative approach facilitated access to the program, normalized the presence of a deaf student, and contributed to an inclusive and non-marginalizing experience. Once the rotation began, the student identified potential barriers to the rotation including having to use a phone for consults, learning new clinical skills under traditional instructional models, responding to codes, and navigating field experiences, all of which could be removed using accessible practices. Each area is addressed below.

## POTENTIAL CHALLENGES FOR DHoH STUDENTS

### Phone Calls

While phone calls in the ED were a challenge for the student, these barriers were easily addressed. For this rotation, the phone was frequently used to access the language interpreting line, consult with the pharmacy, specialist physicians, and the laboratory. To facilitate phone calls, the student used assistive devices including adaptive headsets and video relay service. A speakerphone function or a two-way headset was the chosen method for facilitating phone calls, with the DI on each call interpreting for the student. This was a productive and effective method for removing barriers in this setting. A quick and professional disclosure that the student was using an interpreter or relay service reduced potential confusion when the student’s gender did not match the voice of the DI, or if the receiving party was unfamiliar with communicating with a deaf person.

### Learning Procedural Skills

The acquisition of procedural skills is an essential part of any rotation. Standard EM procedures range from laceration repairs and venipuncture to central line placement and endotracheal intubations. The traditional model of “see one, do one, teach one” whereby students watch a demonstration of a procedure, practice a mock simulation, and then demonstrate competency to a preceptor needed to be modified for the student. Typically, when demonstrating a procedure, the spoken instructions and demonstration often occurred concurrently. For a deaf student, it is difficult to simultaneously focus on both the procedure and the interpreter to capture the instructions. In these situations, the student felt empowered to request that faculty discuss the procedure first, followed by a demonstration of the procedure to allow the student to view the interpreting of instructions before shifting to the demonstration. Allowing time for verbal instruction in advance of demonstration was necessary for the deaf student to have full access to the material. While this approach to teaching the material is necessary for the deaf student it can also increase retention for all students by tapping into multiple learning styles.

### Codes

During a code, communication is essential to ensure role expectations and the team’s approach to the case. When a deaf team member participates in the code they can easily follow their assigned role under the direction of the DI. Additionally, when deaf students become physicians and run a code they can develop strict communication protocols, ensuring that each team member understands designated hand signals. During this rotation, the student and the DI participated in several codes without incident. For each code, interpreter positioning was quickly identified and a line of sight was established to facilitate the student’s involvement and interaction with the code.

### Field Experiences

As part of the rotation, the student was expected to complete a ride-along with emergency medical services (EMS). Excusing the student from field experiences had been the approach during other rotations; however, this program felt strongly that the student should engage in all aspects of the rotation and that the rotation should be fully accessible. The student and the DI were included in required field experiences, including the ride-along in the ambulance. Observing the EMS crew was the main learning objective of the experience. However, the crew was called to an acute incident during the ride-along that necessitated an all-hands-on-deck approach. The student was included in the response by using non-verbal communication (hand signals) and by handing appropriate supplies and pointing or guiding the student’s hands to the needed medical procedure. The DI facilitated verbal communication by establishing a position near the paramedic and emergency medical technician and interpreting essential instructions to the student.

## MECHANISMS FOR ENSURING COMPLIANCE WITH THE ADA

The ADA was amended effective January 1, 2009, and new ADA regulations took effect March 15, 2011.^26^ In the most general terms, the amendments and regulations broaden the definition of a disability, lowering the burden of proof to establish oneself as a person with a disability. The law requires medical education programs, including undergraduate medical education (UME) and graduate medical education (GME) to engage in an interactive process (see Figure) with qualified individuals that includes a discussion about their disability-related needs. This process calls upon disability specialists, program directors and other identified stakeholders to investigate potential and reasonable accommodations that would allow equal access to the program. Appropriately responding to ADA requests for accommodation requires that UME and GME designees maintain a full understanding of federal regulations, are able to articulate the essential functions of their programs and have a command of reasonable and effective accommodations. This case study highlights the effective, respectful, and proactive process among the parties.

## CONCLUSION

A number of methods exist that allow for the full inclusion of DHoH students in medical education including ASL interpreters, DI, Cued Speech transliterators, and adaptive hearing devices. DHoH students with appropriate accommodations, including assistive technology, are able to effectively follow procedural instructions, respond to codes, and respond to other environmental cues effectively, even though these tasks are communication-dependent.

Given the large number of people with hearing loss that affects communication access, it is critical that the growing number of DHoH physicians in the pipeline be well trained and positioned to provide effective, culturally sensitive care. This is especially critical when navigating the communication challenges in EM environments. As evidenced in this case study, the logistical hurdles to access for a deaf student in an EM rotation, and for DHoH students broadly, can be remedied with creativity, advanced planning, and the institutionalization of team-oriented learning environments that prioritize clear communication.^26^ This equips DHoH students to not only effectively handle a complex and diverse patient population, but also increases patient-provider concordance.

## Figures and Tables

**Figure:**
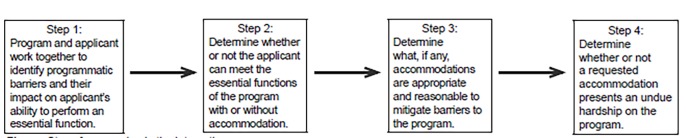
Steps for engaging in the interactive process.

**Table:** Mechanisms for communication with deaf or hard of hearing students.

American Sign Language interpreters (ASL)	A person trained in translating between a spoken and a signed language.
Designated healthcare interpreter (DI)	A designated interpreter is a linguistically specialized sign language interpreter who works extensively with a deaf healthcare professional, making cultural and professional adaptations to the professionals’ career environment as appropriate.
Communication Access Real-time Translation (CART)	A captioner (CART provider) uses a court reporting stenography machine, a computer and software to display everything that is being said, word for word. The text is displayed on a computer, television or projection screen.
Cued Speech Transliterators (CST)	A visual mode of communication that uses hand shapes and placements in combination with mouth movements and speech to make the phonemes of spoken language visible.
Video Relay Service (VRS)	Video Relay Service is a form of Telecommunications Relay Service that enables persons with hearing disabilities to utilize ASL to communicate with voice telephone users through video equipment, rather than through typed text. Video equipment links the VRS user with a TRS operator – called a communications assistant, or CA – so that the VRS user and the CA can see and communicate with each other in signed conversation.
Adaptive hearing devices	A device that helps individuals with hearing loss or a voice, speech, or language disorder to communicate. (examples: Induction loops systems; FM systems, infrared systems; personal amplifiers, amplified stethoscopes, digital stethoscopes).

## Footnotes

* Hearing loss throughout this article is defined as mild (>25 dB–40dB), moderate (>40dB–60dB), severe (>60dB–80dB), and profound (>80dB).
